# Service Users’ Perspectives on an Integrated Electronic Care Record in Mental Health Care: Qualitative Vignette and Interview Study

**DOI:** 10.2196/64162

**Published:** 2025-06-03

**Authors:** Timothy Kariotis, Megan Prictor, Kathleen Gray, Shanton Chang

**Affiliations:** 1 Faculty of Engineering and Information Technology, University of Melbourne Carlton Australia; 2 Melbourne Law School, University of Melbourne Carlton Australia; 3 Centre for Digital Transformation of Health, University of Melbourne Parkville Australia

**Keywords:** electronic health record, mental health care, qualitative vignettes, mental health, social care, qualitative study, user perspectives, health care, Australia, semistructured interview, service user, stigma, health record, patient portal

## Abstract

**Background:**

There have been suggestions that electronic health records (EHRs) should be expanded beyond clinical mental health care services to a broader array of care services that support mental health service users, which we call an integrated electronic care record (IECR). Previous research has considered service users’ general views on information being stored and shared via an EHR. However, little consideration has been given to service users’ attitudes toward how EHRs should be used in the provision of care or the concept of an IECR.

**Objective:**

This study aimed to understand mental health care service users’ perspectives on an IECR and how it should be used in practice when receiving care.

**Methods:**

Ten people with lived experience of accessing multiple services in Australia’s mental health care system were provided with 2 vignettes that depicted fictional service users making decisions about an IECR. Participants were asked to respond to several scenarios that the fictional service users might experience in their journey through the mental health care system with an IECR. Participants provided written responses and took part in a semistructured interview to discuss their responses. An interpretative phenomenological analysis was undertaken, which led to 5 major themes and 15 subthemes being developed.

**Results:**

Service users wanted an IECR that they had control over, supported them as equal partners in their care, and contributed toward more collaborative and proactive mental health care. However, participants were concerned that care professionals’ perspectives would be privileged in the IECR and overshadow service users’ needs. Participants also had concerns that stigmatizing and discriminatory information documented in their IECR would negatively impact their interactions with the mental health care system and their access to care. Participants saw value in an IECR bringing together information to support collaborative and proactive care. However, participants thought that the benefits of the IECR had to be balanced with potential risks to their privacy. Participants thought that the IECR should contain only information relevant to their care and should be shared only with relevant care professionals. There were concerns that service users might lack the skills, resources, and information required to manage their IECR.

**Conclusions:**

An IECR has the potential to fill the gaps in an increasingly complex and fragmented mental health care system but risks entrenching service users’ experiences of stigma and discrimination unless service users are meaningfully involved in their IECR.

## Introduction

### Background

Electronic health records (EHRs) are increasingly adopted in mental health care services to improve information collection, sharing, and use [1]. EHRs promise more integrated and connected care [2-4], especially when they enable information sharing across different care services. EHRs may be especially promising in the mental health care system due to the complex array of services that service users access [5]. Recent attention has turned to how EHRs may enable information to be shared beyond clinical services to include the various social and community care services that people with mental health conditions access [1,6,7]. However, care professionals have raised numerous concerns about the adoption of EHRs in mental health care contexts, including how sensitive information should be documented and the impact of EHRs on the therapeutic relationship [2]. Most of the available research involving service users has focused on their general attitudes toward the electronic storage and sharing of their health data [3], especially secondary uses of EHR data [4]. There has been limited research on service users’ attitudes toward how EHRs should be used in the provision of mental health care, especially EHRs accessible by multiple types of care professionals. We sought to explore service users’ perspectives on how an integrated EHR, which would include clinical and social and community care services, could be used in their care.

Information and how it is managed is critical to modern health care, with the health record being central to providing quality care [5]. Information is managed through a range of information practices of both service users and care professionals, including disclosing, documenting, seeking, and sharing information [8]. The health record plays a key role in these information practices, including enabling communication among care professionals, providing a central source of information about a service user, and acting as an informal workspace [9]. Increasingly, health records are being replaced by EHRs, many of which can be accessed by multiple care professionals and service users [10,11]. Because EHRs are used mainly by care professionals, their experience and needs have been the primary focus of research in the design of EHRs [12-14]. However, there is increasing recognition of how EHRs shape service users’ experiences of care and the need to design EHRs that contribute toward person-centered care [13,15].

### Integrated Electronic Care Record

*EHR* is a broad umbrella term that captures a range of technologies used to collect, manage, and share information about service users [16,17]. Generally, an EHR is an electronic record of patient information that is accessible by care professionals within or across health services. Some EHRs provide service users with access via a patient portal. An increasing number of EHRs seek to collate information from all treating care professionals rather than from a specific service or type of service [18,19]. For example, Australia’s My Health Record is a national summary record that allows any treating clinician to access and contribute information. However, EHRs tend to be limited to health care services and exclude many other social and community care services that play a critical role in mental health care [20].

There is increasing recognition of the need to improve information sharing between all the services that contribute to people’s care, not just those in the health care system [21,22]. In Australia, a recent Royal Commission into Victoria’s mental health care system recommended establishing a state-wide Electronic Mental Health and Wellbeing Record and a Mental Health Information and Data Exchange to facilitate information sharing between all services that contribute to people’s mental health and well-being [23]. This is similar to a recommendation made by the Australian National Mental Health Commission [24] in 2014 that there was a need for an EHR accessible by services beyond the health care system. In this study, the term *integrated electronic care record* (IECR) is used to capture this concept of a digital record that all care services can contribute to and access regardless of whether they are within or outside the traditional health care system.

### Experiences of EHRs in Mental Health Contexts

Care professionals have raised concerns that EHRs will impact the therapeutic relationship and how sensitive information is documented [25]. The concerns regarding the therapeutic relationship relate to how an EHR might impact communication between service users and care professionals [13]. For example, studies exploring the adoption of EHRs have found that they can act as a barrier to communication in clinical encounters [26]. The issue of documenting sensitive information is related to whether such information is appropriate for other health care professionals to view, the perceived risk of sensitive information being shared too widely, and the risk of service users experiencing an adverse reaction when accessing their EHR [2,27].

There is limited research on mental health service users’ attitudes toward EHRs. A recent scoping review of EHRs in mental health contexts found that 10% (4/40) of the studies included a service user perspective. A service user perspective is important because they have different views compared to care professionals regarding how EHRs should be used in mental health care [28]. Previous research has focused on mental health service users’ general attitudes toward the storage and sharing of mental health information via an EHR and electronic health information exchange more generally [3,4,29]. In comparison, research with care professionals has focused on how an EHR is used in practice [25]. Because mental health care involves various sensitive information practices, such as people sharing a range of sensitive and potentially stigmatizing information [30,31], and service users may experience stigma from health professionals [32], the increased availability of information via an EHR may be concerning for service users. There is evidence suggesting that EHRs may affect service users’ decisions to disclose information to care professionals due to privacy and security concerns [33]. However, at the same time, service users may be more likely to want their information shared if they see a practical benefit for their care [3].

We aimed to understand mental health care service users’ perspectives regarding an IECR and how it should be used in practice when receiving care and to answer the following questions: (1) on the basis of service users’ experience of the mental health care system, how do they think an IECR would be used in their care? (2) If an IECR were implemented in mental health care contexts, how would service users want it used in their care?

### Note on Terminology

In this paper, we use the term *care professional* to capture everyone who provides care across the health, mental health, and social and community care systems.

We specifically chose to use the term *service user* rather than terms such as *patient*, *consumer*, or *person*. We acknowledge that each of these terms comes with certain assumptions. For this study, *service users* best captured our intention to focus on people actively engaging with the mental health care system.

## Methods

We used qualitative vignettes and semistructured interviews to answer the research questions by exploring service users’ attitudes toward an IECR. We were informed by the Critical Appraisal Skills Programme’s checklist [34] for qualitative research.

### Context

Australia’s mental health care system is split across several systems and levels of government [35]. Primary health care, including general practitioners and psychologists, are private services that service users receive rebates to access through the federally funded Medicare system. Secondary mental health services, including community mental health care, are funded by state and territory governments. Another critical source of care is the National Disability Insurance Scheme (NDIS), which provides funding for disability support services, including psychosocial support, and sits separately from the health and mental health care system. Other support services such as housing and homelessness services, drug and alcohol services, and community support are provided by the state, territory, and local governments.

### Study Design

We used a qualitative vignette method to answer the research questions. Vignettes are short stories or scenarios involving a fictional individual, which can be used to explore participants’ perspectives on specific scenarios [36,37]. Qualitative vignettes include concrete examples of people, behaviors, and systems and can provide the opportunity to explore people’s interpretation of specific scenarios and sensitive issues or cases in which subjective judgment is required [36,37]. There are various approaches to how participants respond to vignettes [36]. In this study, participants were asked to consider the advice that they would provide to a fictional character in the vignette based on their own lived experience. Vignettes have been used in other studies to explore service users’ attitudes toward and perceptions of EHRs [38].

### Ethical Considerations

The University of Melbourne Human Research Ethics Committee approved this study under application 13697. Participants were emailed details of the study, including a plain-language statement and consent form. Participants were compensated for their time with an AUD $80 (US $51.51) gift card. Written responses, interview recordings, and transcripts were assigned a number, and all identifying information was removed.

### Study Instruments

Two vignettes were developed involving fictional characters and situations designed to reflect common experiences identified in the literature and our previous research on service users’ experience of the mental health care system. Central to the vignettes was the concept of an IECR, with the vignettes including a 1-paragraph description of the IECR (Textbox 1). The description of the IECR was purposefully short to encourage participants to ask questions and identify issues that they would need to consider if an IECR was implemented. A similar approach is taken in the use of technology probes in human-computer interaction research, which are minimally designed to explore how participants see this technology fitting into their lives [39]. Two narratives presented different contextual factors that may resonate more with certain participants. The vignettes and questions are provided in Multimedia Appendix 1, with the key differences outlined in Textbox 2. Participants were provided with a written and audio version of the vignettes and questions that they were asked to provide written responses to before taking part in a semistructured interview. The written responses and interviews were used as a form of triangulation to support the credibility of the results. Participants who struggled to provide written responses to the vignettes were invited to read and respond to the vignettes and questions during the interview.

The interview schedule included 3 sections. First, participants were asked to give their general reflections on the vignettes. Second, specific follow-up questions were asked based on their written responses. The follow-up questions focused on answers in which participants had made assumptions, asked questions, or provided limited detail. Third, general questions were asked about some of the themes identified in the previous literature and in the introduction to this paper regarding how stigma and the retelling of their story should be managed in the context of an IECR.

**Description of the integrated electronic care record in the vignettes.**

**Jona**

Recently, Jona’s general practitioner told him that they had adopted an “Integrated Electronic Care Record (IECR).” This IECR would allow all the services Jona accesses—the GP, homelessness service, psychiatrist, and pharmacist—to access and share information online through a secure internet site. Jona would also be able to access his IECR and the information on it, as well as being able to record information on his record and upload information from personal devices. Services would only need verbal consent from Jona to upload or access information in the record. The GP says that the record could include Jona’s medication record, housing history, consultation notes, test results, and referral letters. Jona’s GP asks him if he would be happy to agree to have an IECR.

**Riley**

Recently, Riley was told by her psychiatrist that an “Integrated Electronic Care Record” (IECR) was being implemented and that she would receive one unless she chose to “opt-out.” The record would allow all her service providers to view her health record and share information electronically. It would also allow Riley to access her IECR and the information on it. Riley would also be able to record information on her record and upload information from personal devices. The psychiatrist says that the record could include Riley’s medication record, consultation notes, test results, and referral letters.

**Summary of the vignettes.**

**Story 1: Jona**

- Opt-in integrated electronic care record (IECR)
- Lives in an urban area
- History of living interstate
- Diagnosed first with schizophrenia and then with borderline personality disorder (BPD)
- Experience of homelessness
- Accesses welfare payments
- No family support and limited social network
- Involuntarily admitted to an inpatient psychiatric ward
- Accesses the National Disability Insurance Scheme

**Story 2: Riley**

- Opt-out IECR
- Lives in a rural area
- Diagnosis of BPD
- Close family support
- Comorbidity of type 1 diabetes
- Voluntarily admitted to an inpatient psychiatric ward
- Experience of substance use issues

### Recruitment and Participants

Our primary population of interest was people with mental health conditions that require them to access a range of health care, mental health care, and social and community care services. This framing may appear broad, but we wanted to avoid specifying a set of diagnoses that we may assume correlate with complexity (eg, so-called severe mental illness) but that people may not identify with and may risk alienating participants. Instead, we chose to focus on the experience of accessing multiple services as it is this experience that an IECR would seek to improve.

Participants were recruited through 2 approaches. First, service users who had participated in our previous research and had consented to be contacted about future research were emailed with details of the study. Second, the study details were circulated to mental health care organizations that included service user advisory groups. The reason for inviting previous participants and service users with experience in advisory roles was due to the effort required to read and respond to the vignettes and to minimize the risk of distress among participants sharing their experiences.

We planned to recruit and analyze data iteratively until we reached data saturation, where similar concepts were reoccurring in the data analysis. We expected that we would reach saturation with fewer participants using the chosen methodology than using other qualitative methods given that all participants responded to the same vignettes. Through our iterative analysis of the interviews, we used an online whiteboard to cluster the codes and concepts from each interview. We determined that saturation had been reached when no new unique clusters of codes were identified.

Ten participants expressed an interest in taking part in this study, and all were recruited to participate. Of those 10 participants, 7 provided written answers to the vignettes before taking part in an interview, whereas 3 provided verbal answers to the vignettes during their interviews. The interviews with the participants who did not complete the prewritten activity were generally longer as the interviewer had to explain the scenarios and the questions. We chose not to collect demographic details of our participants given that our research did not intend to consider how demographics might relate to the qualitative themes. The participants all had a mental health condition and experience accessing a range of care services.

### Analysis

All interviews were transcribed with the support of automated transcription software [40]. We used the inductive interpretative phenomenological analysis methodology from Smith et al [41] to analyze the data from the interviews and written responses. Interpretative phenomenological analysis is a methodology useful in understanding how people’s lived experience shapes their experiences of situations [42]. The first author read each transcript and the associated written response and performed in-line coding using the comment function within a word processor. The research team each coded a subset of the transcripts and discussed their coding decisions to determine the breadth of potential codes. These codes were compiled and organized to identify common clusters of codes within each transcript. Codes and clusters from each participant were uploaded to an online whiteboard and clustered into common groups. These groups were compared with the coding and clusters developed for each participant and discussed among the research team to ensure that each participant’s data were appropriately reflected in the groupings. The research team discussed these clusters until they reached a consensus on how to determine the appropriate delineation and naming of themes and subthemes.

## Results

### Thematic Results

The analysis led to the development of 5 major themes and 15 subthemes, which are outlined in Table 1. These themes were framed based on the participants’ attitudes toward how an IECR *should* be used in their care. However, at the same time, the substance of each theme also reflects how participants expected an IECR *would* be used, based on their experience within the mental health care system. Further illustrative quotes are provided in Multimedia Appendix 2.

**Table 1:** Outline of themes and subthemes.

Theme	Subthemes
Service users have agency and control over their IECR^a^ (theme 1).	Care professionals minimize the use of the IECR during inpatient stays (subtheme 1.1). Service users can establish a trusting relationship with care professionals before their IECR is used (subtheme 1.2). Service users can see how their IECR is accessed and used (subtheme 1.3). Service users have control, through informed consent, over who can access their IECR (subtheme 1.4). Service users have control over what information in the IECR is available to different care professionals (subtheme 1.5).
Service users’ perspectives, needs, and voice are included in what is documented in the IECR (theme 2).	Stigmatizing or discriminatory information is not documented in the IECR (subtheme 2.1). Information in the IECR supports rather than overrides the service user’s needs (subtheme 2.2). Service users can contribute and shape the information in their IECR (subtheme 2.3).
The IECR brings together an accurate record of relevant information about the service user (theme 3).	Care professionals document information in the IECR that balances the need for care and the risk to service users’ privacy (subtheme 3.1). The IECR is a reliable and accurate record according to the service user (subtheme 3.2).
Service users are empowered to confidently use their IECR (theme 4).	Service users receive detailed information about their IECR (subtheme 4.1). Service users are supported to use their IECR (subtheme 4.2).
Care professionals use the IECR to proactively support and coordinate care across all services (theme 5).	The IECR supports collaborative and integrated care across all services (subtheme 5.1). The IECR supports more proactive care and feedback loops between services (subtheme 5.2). The IECR supports service users in retelling their story (subtheme 5.3).

^a^IECR: integrated electronic care record.

### Theme 1: Service Users Have Agency and Control Over Their IECR

#### Overview

Participants thought that service users should be able to manage who can access their IECR and what information they can view and that this required well-designed consent options that provided service users with explicit control over their IECR. The issue of consent was complicated when considering inpatient admissions and the level of agency that service users maintained during periods of acute symptoms. Participants thought that access to the IECR should be based on a trusting relationship between the service user and the care professional. However, in a time- and resource-limited care system, there may be limited opportunities to develop this trust before care professionals need access to a service user’s IECR. In reflecting on how service user autonomy could already be circumvented in the mental health care system, some participants were concerned with how an IECR may be used in such situations. For example, one participant reflected on the fact that, in some circumstances, people with a diagnosis of schizophrenia could lose agency over their finances.

#### Subtheme 1.1: Care Professionals Minimize the Use of the IECR During Inpatient Stays

A question in both vignettes prompted participants to consider how the IECR should be managed in voluntary and involuntary inpatient care scenarios. Five participants thought that access to the IECR should be limited during inpatient care to reduce any distress that service users may experience knowing that their IECR is being accessed, especially without their consent. Participants acknowledged that the information in the IECR could benefit service users’ inpatient care. However, they thought that it should only be accessed with informed consent from the service user or a trusted family member or carer.

Because they were involuntarily hospitalised, because they did not have access to their records, so they couldn’t check their record and see that they were happy with it, that there was right information on there. If they’re not allowed to access their own information, no one should be because that’s just—it feels like a human rights violation to me. Like they should be able to access their record whether they’re well or not. Either with or without a family member present. But that’s their own record and for doctors to be accessing and adding things while that person can’t even see what’s going on, on that record, that’s a big no-no. Talk about trust issues, that’s...you can imagine being unwell, having schizophrenia. Having your voices screaming at you from outside of you. Like “they’re putting down the wrong medications, they’re trying to kill you.”...Yeah, like I couldn’t imagine how I’d be feeling when other people...could access my records but I was not allowed to. That just—just seems really wrong.

P3011

Some participants assumed that, during a voluntary admission, the service user would have access to their IECR, whereas in an involuntary admission, they would not. Participants thought that, if the service user cannot access and manage their IECR during an inpatient stay, which participants assumed would be the case, then no information should be uploaded to their IECR until after they are discharged and with their consent. Some participants considered that information could still be uploaded to the IECR during an inpatient stay but flagged for review by the service user before being made available for other care professionals to view after the admission.

Two participants thought that the record should be accessed and kept up-to-date during inpatient stays to inform the service user’s treatment.

#### Subtheme 1.2: Service Users Can Establish a Trusting Relationship With Care Professionals Before Their IECR Is Used

All participants discussed the role of trust in shaping how service users share information with care professionals, which would also shape service users’ perceptions of the IECR. Participants thought that service users would need to meet the care professionals first to ensure that they can establish a rapport and trust before providing them with access to their IECR. An element of building trust related to theme 2 was that care professionals should get to know the service user rather than just making assumptions about them based on their IECR. The need to establish a trusting relationship was contextualized with comments that Jona and Riley from the vignettes might feel frightened and isolated if they moved locations and saw a new care professional. Participants also questioned whether access to the IECR could be revoked if Jona or Riley found that they could not establish trust or rapport with the care professional.

It depends who you let see it. I’d really have to trust someone...for instance if you go to a psychologist or psychiatrist, I’d have to have a couple of session with them before I knew that I could trust them. Because you have to build that rapport with them before you can let them and trust them with your information.

P3030

Five participants raised the broader issue that service users might not trust the mental health care system or might have developed trust issues from previous life experiences, which may factor into whether they trust the use of an IECR.

#### Subtheme 1.3: Service Users Can See How Their IECR Is Accessed and Used

Five participants discussed the need for transparency over who accessed their IECR, what organization they were from, and what information they accessed or uploaded. Some participants discussed the current lack of transparency over what information is shared between care professionals. Transparency was also viewed as important for managing their symptoms, such as paranoia over who might be accessing their record. Some participants also thought that there needed to be transparency within care encounters as to whether the care professional had read the record and sensitivity regarding how information in the IECR was raised with the service user.

Without them absolutely blurting out, “well I know da-da-da,” maybe...their introduction to you should be, “I have read your record, is there anything you’d like to talk about?”

P3090

#### Subtheme 1.4: Service Users Have Control, Through Informed Consent, Over Who Can Access Their IECR

All participants thought that service users should have control over who can access the IECR and that providing access should be based on informed consent. Participants also questioned whether consent should be continuous (consent is provided for each care professional until the service user revokes it) or episodic (care professionals would have to seek consent each time they wanted to access a service user’s IECR). Participants outlined how Jona and Riley should only consider providing consent to care professionals with whom they have a current and ongoing trusting relationship. One participant recommended that the default IECR setting should be to remove access for care professionals whom they have not seen in a while. There were also questions about how nuanced the consent options would be and whether service users could reveal only parts of their record to certain care professionals. The vignettes explicitly stated that verbal consent would be sought from service users for a care professional to access their record. Two participants questioned how their consent would be documented so they had evidence that they had given it. Some participants questioned whether care professionals could share information in the IECR with other care professionals without service user consent.

...how could we show whether or not I provided consent if I’m not signing anything?...my concerns are around, like Jona has a couple of diagnoses and experiences that are really quite stigmatised and wanting to make sure that Jona is able to protect himself around—like if he wants to access a service but doesn’t want to reveal parts of that...like can I say, my pharmacist can access my scripts, and my mediations, but not my housing information.

P3020

In addition to having a thorough and informed consent process, participants thought that consent preferences should be visible to service users so that they can be confident that they are being followed. Participants identified that a potential barrier to informed consent is when service users are unwell or experiencing symptoms that may impact their ability to consider the benefits and risks of providing care professionals with access to their IECR.

Five participants raised queries about family involvement in the IECR, including how much control service users had over family members accessing their IECR. One participant identified that there may be circumstances in which a family member or another support person might need to access Riley’s record to support them. Conversely, another participant, in reflecting on Riley’s story, considered that, if Riley had a negative relationship with her parents, she might not want to provide access to her family members.

If Riley later has children or a violent partner or a toxic employer or family carer or workers compensation claim, how will you ensure her legal rights, privacy, + confidentiality are protected?

P2050

#### Subtheme 1.5: Service Users Have Control Over What Information in the IECR Is Available to Different Care Professionals

In addition to deciding who can access the IECR, 6 participants thought that service users should have control over what information different care professionals can view. One participant outlined how the onus should be on the care professional to justify to the service users the information they need to access in the IECR. Some participants framed this as ensuring that care professionals can only access what is relevant to their care and information that they will use in providing care. Participants also identified factors that service users might consider when determining what information to allow care professionals to access, including whether the information is accurate and up-to-date, whether they are comfortable sharing it with a new care professional, whether it is relevant for that care professional, and the current reason they are accessing care. Some participants also acknowledged that the IECR may include information from difficult periods in a service user’s life, which they may not want current care professionals to access.

...is there a way for me to block that information...from another service, if I’ve just had a bad day and yelled at someone because I’ve been sleeping on the street for three weeks?...if I move out of this difficult period of my life to where I’m flourishing more, does my new pharmacist need to know that I was also accessing homelessness services?

P3020

The concept of “relevance” was pervasive across several answers that participants provided as to who should be able to access their IECR and what information they should be able to view. These 2 issues intersected in that relevant information may depend on who is accessing it and in what circumstances. For example, one participant thought that, if Jona gave the NDIS access to their IECR, it should only be for information relevant to supporting their application. This issue of relevance is further explored in theme 3.

### Theme 2: Service Users’ Perspectives, Needs, and Voice Are Included in What Is Documented in the IECR

#### Overview

Seven participants were concerned that the IECR would be used as the key source of information in providing care and would override service users’ voice and needs. Underlying this concern was the risk that the IECR may only reflect care professionals’ view of the service user, which may be outdated, include only negative information, and possibly include stigmatizing or judgmental information. However, 2 participants thought that it would be valuable for their care professional to review the IECR before a session so that they have the most up-to-date information. The risk that the information in the IECR will be prioritized over service users’ voice could be managed by including service users’ views and needs in the record (subtheme 2.2) and by ensuring the relevance, accuracy, and quality of the information in the IECR (theme 3).

#### Subtheme 2.1: Stigmatizing or Discriminatory Information Is Not Documented in the IECR

Nine participants raised the risk that an IECR might entrench service users’ experiences of stigma, judgment, and discrimination both inside and outside the mental health care system. Participants were concerned that an IECR would allow for a greater reach and permanency of information that might negatively impact the care they receive, including their ability to access care. Several participants were concerned that information in the IECR might “leak” outside the health care system, such as to employers, and would lead to stigma and discrimination in other areas of life. There were also concerns about how certain information in the IECR could lead to the experience of stigma and discrimination within the health care system. For example, some participants were concerned that certain diagnoses being stigmatized, such as borderline personality disorder and also the label “mentally ill,” may automatically lead to someone being treated differently by certain care professionals. Some participants discussed how they had opted out of My Health Record due to the risk of their diagnosis being shared too widely and negatively impacting their access to care.

I purposely was, in some of my answers, pushing the boundaries back of, well, really...why would this be good for Jona or whoever, you know, the person with the condition who’s got disadvantage and discrimination happening? Why would this be good for them? I guess I got a flavour from...the scenarios that perhaps certain people and agencies would think an electronic record is a really good idea and that it wouldn’t disadvantage...the service user at all. As long as we can maintain security, privacy, confidentiality...they won’t be disadvantaged and I think it’s a really strong underpinning for me about power and language and, you know...the electronic record is just neutral, right, it could be good or it could be bad for somebody but unless we address the actual disadvantage of Jona having a mental health diagnosis and all the other issues that he was facing...I suspect that an electronic record will disadvantage him...because of the underlying inequity he is facing.

P3050

Four participants were concerned that historical information from previous admissions, such as diagnoses and treatments irrelevant to their current care, might lead care professionals to prejudge or overlook their current issues or might impact their access to care. For example, one participant outlined how much of the information documented about them was from when they were at their worst, so future clinicians who access the IECR will only see the negative information that has followed them. This situation may inform a deficit-based rather than a strengths-based approach to care—especially if their strengths and achievements are not documented in the IECR. Another participant shared how their previous eating disorder diagnosis still impacted the care they received today even though they had recovered and no longer met the criteria for an eating disorder diagnosis.

Yeah, so I chose not to opt into the My Health Record, because of my history of BPD...after I stopped meeting criteria for borderline disorder, I just straight up don’t have that issue anymore, I went to ED because of some intense anxiety...they read borderline, and then I was treated that way, instead of treated for what I was actually presenting for, and that was just in my hospital record. I didn’t need other services, like, I don’t think my pharmacist needs to know that I used to have a history of borderline.

P2020

Participants were also concerned that information relevant in one care context could be perceived negatively or be stigmatizing in another context, especially by care professionals with less knowledge of mental health. For example, one participant discussed how their previous drug use may negatively affect their access to mental health care even though it was a historical issue that they had overcome. This issue relates to whether information is relevant to care professionals, as discussed previously and further in theme 3. Participants suggested that care professionals needed to document information in their IECR in a way that was respectful and balanced, with consideration to how the service user may view the information.

#### Subtheme 2.2: Information in the IECR Supports Rather Than Overrides the Service User’s Needs

Six participants were concerned that care professionals who accessed the IECR would focus on information from other care professionals and not on the service user’s perspective. The risk of having information precede service users and shape their care before they can discuss their needs was viewed as entrenching a biomedical model of care in which service users’ individual needs are not recognized or are viewed through a diagnostic lens. Furthermore, participants identified a risk that care professionals may not capture the service users’ perspectives or voice in the IECR, further amplifying the care professionals’ perceptions, ideas, and judgments. Even if the service user could contribute to the IECR, participants were concerned that clinical knowledge would be privileged in the IECR and in how the IECR is used. There were also concerns that the IECR may lead to some health issues, especially physical health issues, being overshadowed by the mental health issues documented in the IECR. Some participants thought that it was important for the service user to be able to tell their story before the care professional accessed their IECR as what is written in the IECR may be framed differently.

He would have the very real problem of clinicians not listening to where he is at and what is happening for him, because they are only going on what others have put in his record.

P3011

#### Subtheme 2.3 Service Users Can Contribute and Shape the Information in Their IECR

Although a number of participants thought that it was important for a service user perspective to be included in the IECR, there was less consideration of whether service users should directly add information to their IECR. One participant, when initially asked about the potential for a service user to contribute to their IECR, thought that it was not necessary or valuable. However, later in the interview, they revisited their response and suggested that it could be valuable to have a service user write up their story. There was also consideration given to the potential for service users to add notes to care professionals’ records that put information in their own words or to identify areas in which they disagreed with their care professionals. One participant thought that there was a risk that service users might change their IECR when unwell, which could negatively impact their care.

Yeah, because then you’ve got more input into it. You could say whether you disagree or agree with the doctor. Yeah, I think it’d be good actually. Sometimes doctors are not—like I just said, they don’t understand [laughs] that they’ve said the wrong thing. So, yeah. If you could put little notes there to say I do not understand—I don’t agree with the way he’s described me, or this was—I don’t see it this way or something. That’ll be good.

P3030

### Theme 3: The IECR Brings Together an Accurate Record of Relevant Information About the Service User

Beyond wanting the IECR to recognize service users’ perspectives and needs, participants also discussed what information care professionals should include in the IECR. The concept of relevance was central to participants’ views on what information an IECR should contain, and it appeared to capture both the need for privacy over certain information and the recognition that information could contribute positively to the care that service users receive.

#### Subtheme 3.1: Care Professionals Document Information in the IECR That Balances the Need for Care and the Risk to Service Users’ Privacy

There was an assumption in the vignettes that the IECR would collate all information about a service user. However, participants were generally supportive of a summary of information being included in the IECR and thought that there should be a standard template or guidance for what should be documented. Participants thought that the information documented in the IECR should be relevant to their care and support future treatment, such as their diagnosis, medication, care plan, evaluation of previous treatment, family medical history, presenting problems, the types of services being accessed, and what participants do to manage their condition. One participant thought that the IECR should not include the personal “chit chat” that might also be shared during the clinical encounter. Participants also discussed that service users would need to determine what to disclose to care professionals that might be uploaded to an IECR and the risks posed by this information being shared. One participant outlined the balance of providing enough information to receive appropriate care but not so much as to create a risk of that information being used against them. For example, it may be appropriate to have information about alcohol and drug counseling in their IECR, but depending on how that information is presented, it could be interpreted in a certain way that does not represent their current needs or presentation.

I think it is important to have things like the diagnosis and the types of medication. Maybe just a little bit of what they do outside that helps them with their condition, especially if they have chronic pain and stuff, is there anything they do to help that. Keep it relatively minimal but succinct at the same time, because I just think that clinicians are honestly just—they’re so overworked that they don’t have time to look through everything. So just keeping it short and sweet and relevant.

P3080

Some participants were concerned that incomplete information could create an inaccurate picture of the service user. These participants were not necessarily in favor of everything being documented in the IECR, with one participant, who had concerns with gaps in their IECR, also stating that only relevant information to support treatment and not personal information should be uploaded. Participants also had questions about what would happen to their information if they opted out of the IECR for a period and then opted back in. In general, participants did not consider that the IECR should be a comprehensive record of all the information from the various services they accessed but that the IECR needed to present an accurate picture of the person’s needs.

...there was another question that I answered stating that if a person opts out and then after six months opts back in, will that record that he had previous be put back on? (Interviewer: Is that important), Oh, yeah, I think so. I think any record is important.

P2010

The issue of sensitive information was only explicitly raised by 3 participants and, generally, was considered as not appropriate to document in an IECR. One participant classed their sexuality as potentially sensitive information, whereas another classed their experiences of hallucinations and delusions as sensitive information.

#### Subtheme 3.2: The IECR Is a Reliable and Accurate Record According to the Service User

One of the scenarios in the case studies was of a care professional watering down information uploaded to the IECR. Three participants were concerned that such a scenario may negatively impact the service user if the IECR does not reflect their care and condition. It was acknowledged that watering down information might be done for a valid reason, such as where information may be triggering for the service user. Participants considered that reducing the level of detail documented should be done in conversation with the service user and for their benefit and that information could be reframed to be shared on the IECR. Watering down information may also impact service users’ ability to access other services, such as the NDIS, if those services rely on the IECR to assess eligibility for access to care. One participant outlined that, in such cases, service users should be able to annotate their IECR if information has been omitted, or the care professional should identify when information has been diluted.

So, something like that, watering that down...no history of being suicidal there, is there? So that automatically puts my life at risk. Just that one scenario...and there can be a gazillion things that watered down, is a safety hazard for the person in question...

P3011

Six participants raised concerns and questions about the management of inaccurate information in the IECR, with some sharing experiences of having inaccurate information recorded in their health record. Participants asked how Jona and Riley could receive support if inaccurate information was uploaded to the IECR and who would be responsible for ensuring that the information was accurate. There were also questions about whether service users could edit inaccurate information or whether care professionals would be required to confirm the accuracy of the IECR with the service user. One participant contrasted the IECR with a referral letter, asking “what’s wrong with a referral letter that you give to the patient, and they read and say, oh, yeah, that sounds right but you missed this, or could you write it like this?” (P3050). Participants were also concerned with care professionals introducing their own subjective judgment into the IECR.

Also, because...it’s done quick and rapid, the chances of an inaccuracy occurring can be high...obviously it impacts their health, it could damage—it could do harm to the client.

P3010

Participants thought that the accuracy and relevance of the IECR should be reviewed to ensure that historical information that is not directly relevant to their current care is not available on the IECR. Participants also thought that care professionals should discuss the accuracy and veracity of the record with the service user to identify information from previous encounters that may not present the service user’s perspective or needs (theme 3). One participant who had a challenging experience of seeking to have incorrect information in their health record updated thought that service users would lack the time and resources to engage with their IECR to address such issues.

### Theme 4: Service Users Are Empowered to Confidently Use Their IECR

#### Overview

Most participants had limited experience with EHRs. During the interviews, 8 participants identified that they had heard of an EHR, mainly Australia’s My Health Record. Of these 8 participants, 1 opted out due to the risk of stigma, 1 noted that they had limited knowledge of My Health Record, and 1 shared that their general practitioner did not upload information to their My Health Record.

Four participants noted that being able to view the IECR could benefit service users by acting as a reference point for their condition, supporting their memory, and facilitating discussions with care professionals. Participants thought that seeing what their care professionals had written would help them engage in conversations about things that they agreed with and areas where they disagreed. There was also an acknowledgment that the digital nature of the IECR could benefit some service users, especially those experiencing homelessness, as they would not have to carry paper documents with them. Some participants thought that the IECR provided an opportunity to check that what was being uploaded to the IECR was correct and would allow them to have conversations with their care professionals about issues that they may not be focusing on or to see what their care professionals thought about them.

...if Jona is sleeping rough...Having something electronically, means that you—if it is cloud based, I don’t have to keep it with me. So, I don’t have to be worried about if it is stolen, and I don’t have to be worried about if my bag gets wet when it rains. Like my scripts aren’t potentially going to be destroyed, or even if a paper script is destroyed, I can be like, no, really, I have been diagnosed—like I have been prescribed these things and pointing to an electronic option.

P3020

Four participants thought that service users might require support when reading their IECR as the content could be distressing and the clinical terminology might be perceived negatively. For example, one participant thought that service users may have an experience of thinking, “is that how they really see me” (P3030).

...before the service provider jots information on the record, knowing that the patient will read it, it’s important that whatever he jots down as a record, he notifies the patient of, if it’s something that may cause fright. For example, a diagnosis of cancer, right, you don’t just put it on someone’s record unless you’ve had discussion with that person about it and is informed about it.

P3010

#### Subtheme 4.1: Service Users Receive Detailed Information About Their IECR

Participants actively engaged with the vignettes and spent time considering the benefits and risks of each scenario. Many of the written answers were framed as questions about the IECR. Participants actively considered the trade-offs of different decisions that the vignettes posed, such as whether to opt in or opt out of the IECR. Although some participants were more supportive of or more against the IECR, most saw reasons why someone might opt in or out.

Can he trust the benefits outweigh his fears and mistrust? Does he feel more confident to tell medical staff himself or would this be easier? Why can’t I or a family member simply tell the doctors? What makes this a better option?

P3011

One way in which the case studies differed was whether the IECR had an opt-in or opt-out model. Six participants thought that it was important that service users be actively supported to make an informed choice as to whether to have an IECR. Some participants considered that service users may not have the opportunity to make an informed choice under an opt-out model, especially if they are unwell when they are required to decide whether to opt out. Participants questioned whether service users can opt in or out whenever they want and what happens to their records during periods in which they opt out.

Yeah, so one of my questions would be, can I opt out, after I’ve opted in? Can I—how do I withdraw consent for someone to access it? Those two things would be pretty key for me.

P2020

Participants raised broad concerns regarding the privacy, confidentiality, and security of the IECR. Some participants considered that there were many unknown risks of adopting an IECR because it is hard to foresee risks that may arise in the future. Participants raised various comments and questions about the security of the IECR and what would happen in the event of a security breach, referencing that there has been a lot of online information theft recently in Australia. Participants wanted to know what support would be available to service users in the case of a security breach. There were also concerns, which were identified in other themes, of potential breaches of privacy and confidentiality through inappropriate use of and access to the IECR by care professionals. Participants considered that service users would need assurances as to the privacy and security of their IECR so that they can confidently use it without worrying about the potential risks of their data being inappropriately accessed.

A breach in the security of IECR could mean serious harm to Riley’s health or current employment or her perspective employment.

P2011

Some participants thought that service users would require someone such as an advocate to explain the IECR and help them make an informed choice about how it is used. A verbal explanation was considered important as written information could be overwhelming. However, such conversations may not be appropriate for care professionals to provide given the time constraints placed on them to provide such information.

#### Subtheme 4.2: Service Users Are Supported to Use Their IECR

All participants identified barriers that service users might face when using their IECR. These barriers included the skills needed to manage IECR settings, the resources needed to access the IECR, and the knowledge needed to understand the IECR and the information documented in it. The lack of access to appropriate information technology may limit services users’ ability to use their IECR. Participants questioned whether training would be provided to support service users to utilise their IECR. Participants also suggested that the IECR should be easy to use. For example, password management should be simple because service users may struggle to remember passwords. Several participants questioned what support would be provided alongside the IECR to ensure that service users can use it and manage any issues arising from the IECR, such as inappropriate use by care professionals.

Firstly the “patient log-in password” should be one easy to remember by the patient and hard to decipher by anyone else foreign to the patient...It should not be shared by anyone.

P3010

A couple of participants considered that service users’ symptoms may act as a barrier to using and making informed decisions about their IECR and that this would need to be considered in the design of the IECR and the support available to service users. A couple of participants considered that service users may negatively react to what is written in their IECR but that this should be managed by the care professional notifying the service user before uploading information to the IECR.

Although participants considered privacy settings an important component of the IECR, 3 questioned whether all service users would have the skills and knowledge required to use these settings. Participants thought that in-depth information and support would be required to ensure that Jona and Riley could use and understand the implications of the privacy settings of their IECR. There were questions about who Jona and Riley could contact for support and whether they could access free legal advice if there were issues or breaches with their IECR. Participants acknowledged that, in the vignette, Jona used a public computer to access his IECR, which could raise privacy risks for him.

I would be very selective with my...privacy...whereas in this situation of the two people in the stories, I don’t know if they had enough knowledge and ability to perhaps do that, to work out what amount of privacy they would need.

P3090

### Theme 5: Care Professionals Use the IECR to Proactively Support and Coordinate Care Across All Services

#### Overview

Participants discussed how the IECR could support more collaborative and integrated care across various services. Integrating various sources of information in the IECR may also enable more proactive care by providing care professionals with a more holistic picture of service users’ needs. One participant thought that the IECR might help them prove their eligibility for other services. However, some participants questioned whether care professionals would use the record or whether they would still have to repeat their information when accessing a service.

#### Subtheme 5.1: The IECR Supports Collaborative and Integrated Care Across Relevant Services

Six participants considered that the IECR may support more collaborative care by linking the different care professionals supporting service users. It was acknowledged that the IECR may benefit services that are not usually kept informed, such as pharmacists and the NDIS.

Having One Record...One File...Electronically kept...and shared by everyone Jona agrees to doing with...is better than having many records scattered everywhere among service providers...better collaboration can occur...an easier / more precise healthcare can be administered.

P3010

Although some participants saw benefits in integrating their information via the IECR, 8 were also concerned about the IECR being accessed or information in the IECR being shared with too broad a range of organizations. This concern included whether noncare services such as employers, other government agencies, and the police could access the IECR. Participants also raised the concern that, depending on the policies of different organizations, providing consent for an individual might allow others within that organization to view their IECR. Different participants usually identified a different list of organizations that they were concerned would access their IECR. One participant framed organizations that should not have access as those that did not have the service user’s health as their primary focus. However, one participant considered the NDIS as an inappropriate service to be provided with access to their IECR.

#### Subtheme 5.2: The IECR Supports More Proactive Care and Feedback Loops Between Services

Four participants thought that care professionals might be more informed about the current needs of service users, including their social needs, by having more services involved in the IECR. For example, one participant thought that, in Jona’s story, the IECR may help care professionals identify a risk of social isolation when he moved to a new suburb. Identifying this risk to Jona could prompt care professionals to support Jona in settling into his new community. Including social and community care services was viewed as important by participants because these services tend to have a long-term recovery focus and might see more of the service user over their recovery journey. Participants also identified that bringing together different services on the IECR could support the identification of inconsistencies, inadequacies, or overlaps in care that could, in turn, improve the effectiveness and adequacy of care. Some participants also thought that the IECR might help care professionals link the dots between different pieces of information to identify patterns or other factors, such as socioeconomic factors, that might contribute to their mental health.

IECR also might pick up on Jonas “pattern of unwellness,” noting that Jona tends to have particular problems at certain times of the year so preventative measures and extra supports could be put in place ahead of time.

P3011

Providing a broader range of care professionals with access to the IECR was also viewed as supporting holistic care. For example, one participant thought that having their housing risk assessment and diabetes information in the IECR could better inform other care professionals of their needs.

#### Subtheme 5.3: The IECR Supports Service Users in Retelling Their Story

Nine participants discussed how the IECR could help bring service users’ story together in one place to minimize the need to unnecessarily retell it when accessing new services. Participants thought that the IECR may also make transitions between services easier and provide care professionals with a picture of service users’ current needs. Some participants noted that service users need to share a lot of information just to be prioritized when accessing a service, but at the same time, some of this information can be challenging to share. With the appropriate controls in place, the IECR could support service users in sharing their story, and as one participant put it, make sure that it is “translated to clinical speak.” The IECR could also act as proof for the service user when sharing information with new care professionals. Participants discussed that, when they are acutely unwell, it can be challenging to remember information and put it in words that care professionals will understand. However, as was outlined in previous themes, there is a balance between the benefits of not having to retell the story and having the IECR override the service user’s voice. Some participants were also concerned that care professionals would not read the IECR and they would still be required to retell their story.

Yeah, it can get depressive, it can get frustrating, it can get upsetting because you’re talking about the past and no one wants to go to the past particularly when it’s been related to an unfortunate experience.

P3010

## Discussion

### Principal Findings

#### Overview

This study sought to explore mental health service users’ perspectives on an IECR. The results suggest that service users want to feel empowered to shape the content of their IECR and how it is used. However, participants were concerned that the IECR would likely be used to privilege the perspectives of care professionals, especially if service users were not supported to use their IECR. Participants thought that an IECR could bring together a comprehensive picture of their needs and support more collaborative and proactive care. However, there was also the risk that inaccurate and out-of-date information would overshadow the actual needs of service users or that care professionals would prejudge them based on the information in their IECR. The concept of relevance, including relevant care professionals accessing the IECR and relevant information being uploaded to the IECR, was central to participants’ views on how the IECR should be used. The findings of this study provide insights into how IECRs and EHRs should be designed for use in the mental health care context.

There were some contradictions in the findings of this study. For example, participants wanted the IECR to minimize the need for them to retell their story while also not wanting to lose control of how their story is told. Similarly, participants only wanted relevant information in the IECR but were concerned about gaps in their record. One way to understand these contradictions is in the context of Australia’s mental health care system, where there is an entrenched power imbalance between service users and the mental health care system that contributes to the disadvantage and marginalization of people with mental health conditions [43,44]. Our findings suggest that participants are concerned that an IECR might entrench many of these existing issues in the mental health care system. In the ideal care system, service users might feel confident relying on an IECR as a complete record of their story. However, in the current mental health care system, the risk of judgment, stigma, poor treatment, and a lack of service user control over their care may underpin a lack of trust in how an IECR would be used.

#### Risk of Care Professionals Prejudging Service Users Based on Their IECR

Participants were concerned that the IECR would entrench experiences of stigma and discrimination. This finding is not unexpected given the evidence that people with mental health diagnoses experience stigma and discrimination across various health care settings [45,46]. Stigma operates at different levels in the mental health care system, including the structural (eg, discriminatory policy and social structures), interpersonal (eg, prejudice and misinformation), and intrapersonal (eg, negative beliefs about the self) levels [45,47]. Henderson et al [45], in reviewing the evidence on stigma in mental health care settings, found that research has focused on interpersonal stigma, with less consideration being given to how structural stigma may shape interpersonal stigma. There could be an opportunity in the design of EHRs to manage structural stigma by shaping how organizations collect and manage stigmatizing information, which contributes to interpersonal stigma. Addressing stigma is important as it can act as a barrier to help seeking [48]. Grando et al [49] found that stigma was a key reason mental health service users might not consent for care professionals to access their EHR. Service users also anticipate stigma based on their past experiences, which may shape how they interact with care professionals and the information they disclose [46,50]. Some participants in this study anticipated, based on their previous experiences, that the IECR could further entrench stigma and discrimination and that stigmatizing information may follow them via their IECR.

#### Relevant and Sensitive Information

Participants did not support the IECR being a comprehensive record but rather valued a record that could provide relevant information to relevant care professionals to support their current care needs. This focus on relevance was framed as ensuring that the information available could be used by care professionals to improve their care. This finding supports previous research by Shen et al [3], who, in interviewing mental health service users about their privacy perspectives, found that they were supportive of health information exchange when it would contribute to better care. This finding also reflects modern theories of privacy, such as the theory of privacy as contextual integrity by Nissenbaum [51], which situates people’s experiences of privacy within specific contexts. Nissenbaum [51] frames privacy as the *appropriate flow of information in a specific context*. Thus, some information may not raise privacy concerns when shared in some care contexts, but in others, it may be seen as inappropriate. Thus, the context in which the IECR is used will shape what service users may define as relevant to be shared.

These findings also challenge the purpose of the health record. Historically, the health record was a record of the care that someone received, usually within one specific health care setting [9]. Although EHRs are regularly framed as “digitizing” the health care record [52], EHRs introduce fundamentally different information-sharing practices compared to paper health records [25,53]. Currently, if information is shared between services, it is done via a letter, phone call, specific shared care plan, or the service user. In all these cases, the information shared is curated for a specific point in time, for a specific receiver, and usually as an outcome of the service user engaging with a service [53]. This approach to information sharing has been described as “pushing information” to other services [53,54]. In the case of the IECR or Australia’s My Health Record, information is “pulled” out of a repository with limited curation by another care professional or the service user for that specific receiver [53,54]. There is a risk that an IECR would become unwieldy due to the amount of information stored in it, which risks becoming quickly out-of-date or irrelevant. It may be more appropriate for an IECR to operate similarly to a shared care plan, which is designed to be used in team-based care for a particular condition and include information relevant to the team and the care they are providing [55].

A comprehensive IECR may also breach modern privacy principles such as data minimization and privacy by design. Privacy by design asserts that the minimum necessary information should be collected for a specific purpose [56]. The challenge with IECRs, as articulated in this study, is defining what is useful or relevant at the point of collection in a complex care system in which the next step in someone’s care journey is not always clear. The risk of an IECR is that it goes in the opposite direction of data minimization toward data maximization, which poses practical issues for care professionals trying to find relevant information and privacy issues for service users. Even when EHRs allow service users to place access controls on parts of their record, this may not be feasible when there are large amounts of content in the record.

#### Managing Sensitive Information

Only some participants in this study raised the issue of how sensitive information is managed. Other studies have found more explicit views on the management of sensitive information. Soni et al [57], in a mixed methods study with behavioral health patients, found that most (76%) participants considered mental health information sensitive and 24% feared stigma and discrimination in relation to their mental health. Participants also reported wanting to restrict the sharing of mental health information in the EHR. Similarly, a survey study by Soni et al [58] found that participants were less willing to share information that they perceived as sensitive. Mental health information was considered the most sensitive, and many participants wanted to restrict access to some or all of this information by care professionals. This study does highlight that service users consider information collected about them during involuntary inpatient treatment as requiring careful consideration before it is made available to other care professionals.

#### Service User Control and Consent

Consent was a key issue raised by participants, who thought it was important for informed consent to be required before care professionals can access their IECR. However, there are questions as to how consent is managed in cases of involuntary treatment, where service users are deemed to lack the capacity to make decisions about their care. Participants were concerned with how an IECR would be used in such cases and generally were against the IECR being used in involuntary inpatient care. There is growing research on the use of dynamic consent mechanisms and psychiatric advance care plans that would provide service users with the ability to predetermine their preferences for care when they may be assessed to lack capacity [59]. However, research on psychiatric advance care plans has found low completion rates due to barriers such as service users not understanding the advance care plan, the complexity of completing the forms, and skepticism about their benefit [60,61]. Similarly, care professionals report barriers to using advance care plans due to a lack of access to and time to review documents in a crisis and a lack of training [61]. Research on advance care planning has questioned their ability to improve care when the processes required to establish and use an advance care plan rarely align with the realities of clinical practice [62]. There is a broader question as to whether service users should lose their ability to make decisions about their IECR in inpatient settings. There is recent evidence suggesting that most service users with psychiatric conditions in inpatient care have the capacity to make complex and important decisions about their care [63].

Participants also wanted control over what information certain care professionals could access. Given the findings of this study and others [64], it appears that service users would likely want to hide information that they perceive as sensitive or irrelevant to their care. However, Schwartz et al [65], in a study of how service users manage the privacy functions of their EHR, found that most service users who chose to limit access to their EHR chose to limit access to the entire EHR. This finding may be due to the effort required to restrict access to certain types of information [65]. Further research is required to understand how service users make decisions to use privacy controls and approaches to supporting them in managing their IECR. One approach to addressing this issue is through identifying sensitive information upfront when information is documented and allowing service users to preset conditions for when sensitive information can be accessed. For example, Chivilgina et al [66] found in a qualitative study with psychiatric service users that they wanted care professionals to obtain purpose-related informed consent when documenting sensitive information in their EHR.

#### Service User Contributions to the IECR

Participants thought that it was important that service users’ perspectives were captured in the IECR. However, there were concerns that service users may not have the resources and time to independently add information to their IECR. Approaches such as collaborative documentation may facilitate care professionals working with service users to agree on what should be documented in the IECR [67]. However, the design of EHRs may also actively limit care professionals’ ability to capture service users’ stories and perspectives. One of the proposed benefits of EHRs is that they improve data quality and enable the collection of structured and standardized data that can be used for other purposes, such as research. Our previous research [25] and that by Varpio et al [68] have found that EHRs that require structured data entry can limit the capturing of narrative information, which may include service user perspectives. Furthermore, Rathert et al [26], in reviewing the literature on EHRs, found that they may limit the documentation of psychosocial and emotional information. Finding ways to capture, share, and use narrative data appears key to ensuring that service users’ voices are considered in care encounters when using an IECR.

Participants questioned the level of support that service users would be provided with to access and manage their IECR. There were concerns that service users, including the fictional characters in the vignettes, would lack the skills and knowledge to make informed choices about their IECR. Ensuring the usability of EHRs is an ongoing issue in Australia, with research identifying gaps in the usability of My Health Record, including the educational resources available for service users, which may negatively impact those with low health literacy [69-71]. A notable finding in this study was the importance that participants placed on having support to engage with the privacy functions of the IECR. While existing literature has focused on the availability of privacy functions in EHRs [58,72], participants in this study highlighted the importance of having skills and capabilities to manage these functions, as well as support pathways outside the IECR to address privacy issues.

#### Inclusion of Social and Community Services

In presenting participants with the concept of an IECR, we purposefully expanded the potential array of care services that could contribute to the health record, which in turn may expand the types of information available. There is growing research on the inclusion of social and behavioral data in health records, with some researchers positing that such information provides broader context to tailor interventions and clinical decisions [73,74]. One of the challenges that has been identified in collecting such information is that service users may perceive it as sensitive and, therefore, not disclose it [75]. Where care professionals record this information, there is a risk that service users may perceive this negatively, especially if the information is viewed as a pejorative assessment of them, such as assessments of their income. What this study found is that service users perceive such information as enabling more proactive care but are concerned as to how broadly their IECR might be shared. This aligns with suggestions in the literature that service users should be engaged as partners in the process of collecting such information, with transparency over how it will be collected and shared [75].

#### Use of Qualitative Vignettes

The findings suggest that qualitative vignettes are a feasible approach to exploring service users’ perspectives on digital health technologies that they have not yet experienced. One potential avenue for future research using this methodology includes exploring story completion methods alongside vignettes to encourage participants to explore ways in which the vignettes might evolve based on different responses to the scenarios [76].

### Limitations

This study is limited by the breadth of our recruitment strategy. Due to the novel nature of the research topic, we determined that our recruitment strategy would focus on people with a specific experience (accessing multiple services for their mental health) rather than a targeted demographic of service users. Future research could include specific groups of service users, such as those with certain diagnoses, to determine how perspectives on an IECR differ across various groups of service users. The sample is also not representative of all people who access the mental health care system. Those with the most acute presentations were likely not represented in this study due to the approach to recruitment and the resources required to participate. However, some participants did share that they had previous experiences of acute illness, including inpatient admissions.

### Conclusions

This study found that mental health service users see two alternative ways in which an IECR might impact their care. The first is one in which the IECR entrenches issues that service users experience in the mental health care system and adds a new, burdensome system for care professionals to try to use. The alternative is an IECR that contributes to care professionals having access to relevant information that supports collaborative and proactive care and service users having control over and being able to see how their information is managed. Although an IECR will not solve the systemic issues present in Australia’s mental health care system, it could help improve the coordination and proactiveness of care, amplifying service users’ needs and supporting service users as active participants in their care. For an IECR to have a positive contribution to broader mental health care reforms, the design and implementation of such a record should consider how service users and care professionals will be supported in using the record, issues of service user consent, what information is necessary to support coordinated and proactive care, and how service users’ self-defined needs can be captured and acted upon in an IECR.

## Appendix

Multimedia Appendix 1 Qualitative vignettes.

Multimedia Appendix 2 Illustrative quotes.

## Abbreviations

EHR: electronic health record
IECR: integrated electronic care record
NDIS: National Disability Insurance Scheme
