# Supplementary material for: Service Users’ Perspectives on an Integrated Electronic Care Record in Mental Health Care: Qualitative Vignette and Interview Study
Source: JMIR Med Inform. 2025 Jun 3;13:e64162. doi: 10.2196/64162 (PMC12174869; doi:10.2196/64162)
Supplement: Multimedia Appendix 1 [file medinform_v13i1e64162_app1.docx]

**Appendix 1: Qualitative Vignettes**

**Story: Jona Part 1**

Jona is a 46-year-old man who lives in North Melbourne. Jona moved from Townsville to Melbourne when he was 22. Jona enjoys fishing and watching soccer.

Jona has experienced several admissions to inpatient psychiatric facilitates since childhood. Initially he received a diagnosis of schizophrenia in Townsville, but when he moved to Melbourne his new psychiatrist changed his diagnosis to Borderline Personality Disorder (BPD). Over the past several years, Jona has experienced some months of homelessness. He has relied on homelessness services and emergency housing. Jona has a caseworker who supports him to manage his housing. He also accesses Centrelink to receive a Newstart payment. Jona does not have any family to support him and only a small social network.

Jona accesses several services, including a general practitioner, homelessness outreach service, psychiatrist, and community pharmacist.

Recently, Jona's general practitioner told him that they had adopted an 'Integrated Electronic Care Record (IECR)'. This IECR would allow all the services Jona accesses – the GP, homelessness service, psychiatrist, and pharmacist - to access and share information online through a secure internet site. Jona would also be able to access his IECR and the information on it, as well as being to record information on his record and upload information from personal devices. Services would only need verbal consent from Jona to upload or access information in the record. The GP says that the record could include Jona’s medication record, housing history, consultation notes, test results, and referral letters.
Jona's GP asks him if he would be happy to agree to have an IECR.

Jona decides to opt-in to the IECR. This means Jona now has an IECR that all services can access and share information on. Currently, Jona does not have access to a computer, so he relies on a local library to access the internet. He goes to the local library and logs-in to his IECR. He is given the option to choose who can share information on his IECR, what types of information can be shared on the IECR, and who can access what information.

**Questions: Jona Part 1**

**Question 1**: What type of questions do you think Jona should ask the GP before deciding whether to opt-in to the IECR?

**Question 2**: What is your opinion on an integrated electronic care record for Jona? What might be the benefits and risks of such a record?

**Question 3:** What should Jona consider when deciding on the privacy settings for his IECR – including who can access it, and what information is shared on it?

**Story: Jona Part 2**

A year after initially accessing the integrated electronic care record (IECR), Jona is involuntarily admitted to a psychiatric ward for a few months. During this time, Jona is experiencing high levels of distress and is not allowed access to a computer.

A few years after signing up for the IECR, Jona is offered a public housing unit. However, the housing unit is in Newport, which is nearly 30 minutes by public transport from Jona's current suburb. This means Jona is going to have to access different health services in Newport.

Jona starts accessing a new psychiatry service in Newport. When Jona calls to make his first appointment with the new psychiatrist, the receptionist asks if they can have access to Jona's IECR so the psychiatrist can read it before the first appointment.

A few years after moving to Newport, Jona's GP recommends he apply for the National Disability Insurance Scheme (NDIS) to get more support. Jona is assigned a caseworker from a local disability service who will help him through the NDIS assessment process. Jona's caseworker asks if they can access his integrated electronic care record to support the NDIS assessment process.

**Questions: Jona Part 2**

**Question 4:** When Jona is involuntarily admitted to the inpatient psychiatric ward, what should happen to his IECR?

**Question 5**: What might Jona expect to experience when he moves to Newport and starts accessing new services? What role might the IECR play?

**Question 6**: What Should Jona consider when deciding whether to give his new psychiatrist access to his IECR before his first appointment?

**Question 7:** What should Jona consider when deciding whether to give the NDIS access to his IECR? **Question 8:** Do you have any other thoughts about any of the question or topics in this story?

**Story: Riley Part 1**

Riley is 26 and lives in Goulburn NSW, working a casual receptionist job. Riley has lived in Goulbourn since she was 14 when her family moved from Sydney.

Since her early 20s Riley has been living with a diagnosis of Bipolar Disorder and has experienced several stays in inpatient psychiatry. Recently, Riley has been relying on her GP, psychologist, and a psychiatrist. Riley is close to her family and has relied on them in the past to support her when accessing mental health services. In addition to Bipolar Disorder, Riley has lived with Type 1 Diabetes since she was young. Riley self-manages her Diabetes with support from her GP and local primary health nurse.

Recently, Riley was told by her psychiatrist that an 'Integrated Electronic Care Record’ (IECR) was being implemented and that she would receive one unless she chose to 'opt-out'. The record would allow all her service providers to view her health record and share information electronically. It would also allow Riley to access her IECR and the information on it. Riley would also be able to record information on her record and upload information from personal devices. The psychiatrist says that the record could include Riley's medication record, consultation notes, test results, and referral letters.

The psychiatrist ensures Riley that she will have complete control over the record, and she can also give her parents access to the record. Riley decides not to opt-out, which means she will now have an IECR.

**Questions: Riley Part 1**

**Question 1**: What type of questions should Riley ask the GP before deciding whether or not to opt-out of the IECR?

**Question 2**: What is your opinion on an integrated electronic health record for Riley? What might be the benefits and risks of such a record?

**Question 3:** What decision should Riley consider when deciding on the privacy setting for her IECR – including who can access it and what information is shared on it?

**Story: Jona Part 2**

A few months later, Riley decides to look at her record through the online site that allows people to view their IECR. When Riley logs into the record, she is asked whether she wants her data to be used for 'secondary uses' such as academic research. Riley also looks at the information her clinicians have uploaded. She notices that the information is vague and lacking in detail. At her next appointment, Riley asks her psychiatrist why her record was lacking in detailed information. Riley's psychiatrist admits that they 'watered down' the information so that it did not worry her, and because it might not be appropriate for other clinicians to read.

A few years after initially accessing the IECR, Riley is voluntarily admitted to a psychiatric ward for a couple of months. During this time, Riley is experiencing high levels of distress and relies on her family to support her in making healthcare decisions.

Riley has historically struggled to manage substance use. After leaving the psychiatric ward, Riley starts using substances again. Her GP refers her to the local alcohol and drug service, which is linked to the local hospital. The drug and alcohol service asks Riley if she has an integrated electronic care record, they could access.

More recently, Riley’s psychologist – who Riley sees fortnightly – has announced she is moving interstate. Riley reaches out to another local psychologist to organise an initial appointment. The new psychologist asks Riley if she has an IECR they can access.

**Questions: Riley Part 2**

**Question 4:** How might Riley react to her psychiatrist admitting to recording less detailed information in Riley's IECR?

**Question 5:** When Riley is voluntarily admitted to the inpatient psychiatric ward, what should happen to her IECR?

**Question 6**: What should Riley consider when deciding whether to provide the Drug and Alcohol service with access to her IECR?

**Question 7**: What should Riley consider when deciding whether to provide the new psychologist with access to her IECR?

**Question 8**: Do you have any other thoughts on this story of the questions asked?
