# Supplementary material for: Service Users’ Perspectives on an Integrated Electronic Care Record in Mental Health Care: Qualitative Vignette and Interview Study
Source: JMIR Med Inform. 2025 Jun 3;13:e64162. doi: 10.2196/64162 (PMC12174869; doi:10.2196/64162)
Supplement: Multimedia Appendix 2 [file medinform_v13i1e64162_app2.docx]

**Appendix 2: Illustrative Quotes**

| Themes and sub-themes | Illustrative quotes |
| --- | --- |
| 1. Service users have agency and control over their IECR. | *…it kind of implies that…it's automatic unless you opt out…and I can imagine GPs and clinicians going, hey, look, this is easier for us, we get to talk to each other directly and cut you out. So - and in many instances, that's how it will actually work for a lot of people…and a lot of clinicians will be preferring to do it that way rather than actually deal with their client…I feel that there will be pressure on people to just concede and have the record. [P3011]* |
|  | *Health professionals should be able to access it if consent is given. However, Jona should have the ability to deny access at any time. [3080]* |
| 1.1: Care professionals minimize the use of the IECR during inpatient stays. | *Yeah, I think that since – if he's an inpatient…he's not able to have a look at what documents have been uploaded, and what the contents of those documents might be, so I think that there should be a special area of Jona's IECR that would contain any documents that were added to his repository while he was an inpatient. So, they would be kept separate from his pre-existing records... it would remain in that part of the repository…until he’d had a chance to look at it…it would be up to him to move locations of the data that got added while he was an inpatient… back into the location with his other pre-existing documents. [P3070]* |
|  | *…no matter what happens while he’s on ward, he should have the option again, once he’s discharged and had that follow up to either consent, reconsent or withdraw his consent, and that that should be done, whatever he’s decided at that follow up is what should be the thing that goes forward. [P3020]* |
| 1.2: Service users can establish a trusting relationship with care professionals before their IECR is used. | *We couldn’t trust anyone from the abuse we had as children. When you can’t trust your parents, you can’t trust anyone. So, putting things out there digitally that's really personal information, that really is scary for a lot of people...Because we're just - like, you know, can someone break into that information? Would a future prospective employer, once I'd stabilised myself and was well, could they access it? Could a family member access that without my permission? Or, you know, what could go wrong with that? How secure is it really? [P3011]* |
|  | *I think it may be best for Riley to see the Psychologist a few times...get to know him better…then offer to provide her previous records... [P3010]* |
| 1.3: Service users can see how their IECR is accessed and used. | *Yeah, I think maybe you could somehow see who’s logged in and seen your information so you could keep a track yourself. [P3030]* |
|  | *I would want to be able to look at, if possible, if my voices were saying that they'd accessed my health records, or MyGov records, I'd like to be able to look at and access this, and make sure the voices were lying to me. [P3070]* |
| 1.4: Service users have control, through informed consent, over who can access their IECR. | *If she decides to remove her parents, is that an easy process or lengthy one? Also, can she limit how much information her family can see? [P3080]* |
|  | *So, if I had given consent for my recruitment company to look at my medical records, to verify that I had a genuine disability, I wouldn’t necessarily want them to be able to find records for my drug and alcohol counselling. [P3070]* |
| 1.5: Service users have control over what information in the IECR is available to different care professionals. | *I would be more comfortable if I had the power to say, right, I don’t really want you guys to know about this or this or this in my past or in my history. It’s not relevant to what we’re doing now. [P3090]* |
|  | *Question 3: What should Jona consider when deciding on the privacy settings for his IECR – including who can access it, and what information is shared on it?*  *Answer:*  *How much each service should be allowed to access*  *Things he wants to keep private*  *Things he feels he doesn’t want certain services to access. [P3090]* |
| 1. Service users' perspectives, needs, and voice are included in what is documented in the IECR. | *…regardless of the doctor’s opinion that’s in the moment, this person knows their history, knows their diagnosis and their treatment. You know, a lot of people will present to hospital and the doctor will give them a medication that has been proven to be really bad and actually no good for them. They’ll insist on putting on them - on this medication when they’re clearly telling them no, look, this is what I’m on and it’s working for me. Please don’t put me on that. [P3011]* |
|  | *Question 1: What type of questions do you think Jona should ask the GP before deciding whether to opt-in to the IECR?*  *Answer:*  *…. How will my knowledge and experience be recorded correctly?... [P3050]* |
| 2.1 Stigmatizing or discriminatory information is not documented in the IECR. | *Participant: …I got kicked out of NA [narcotics anonymous]…because of my mental health, and I think that if my drug counsellors became aware of that problem, I'm not sure what they would write, and I wouldn’t want my recruitment consultant looking at records, when I can’t anticipate what the record is going to contain.*  *Interview 1: Okay, okay. So, your perception is that if that information was on there, that could reflect badly in other contexts.*  *Participant 3070: Yeah, correct. [P3070]* |
|  | *I have purposely moved out of area to other places and not told GPs my mental health history because you know what, I’ve had, so many times I go to the GP for an earache…or something really clearly physically and they just want to talk about, am I still on lithium, when did I have my last blood test, you know, yadda yadda, and whatever I say, they don’t actually believe. [P3050]* |
| 2.2: Information in the IECR supports rather than override the service user’s needs. | *…they read a bit of the history and come into the room with assumptions so I think approaching people with a needs based conversation – what is it that’s happening right now and what do you need, might actually generate more useful information for both parties. [P3050]* |
|  | *Well, I guess I'm saying that really the medical professional knowledge is privileged over lived experience, and even if Jona is able to access his electronic record and make entries into it, that I'm very cynical about his documentation being held in the same regard and actually as powerful to be as powerful in terms of how he would be treated and his decision making and. A sense of agency and things like that. [P3050]* |
|  | *I don’t know if that was just through my personal opinion, that some services, in reading through all your history and that kind of thing, before they even meet, you can form an opinion. [P3090]* |
| 2.3 Service users can contribute and shape the information in their IECR. | *…that should be taught to them immediately that rule number one, you ask permission and you go through that record with that person. Check in and are you happy with this? Is this accurate? Are there any drugs on here that you had a bad reaction to or any treatments that did or didn’t work for you? You know, all those types of things. If there’s no discussion, well I’d never be having the record. There’d be no way because I’d be like - and not just that, I would get up from that clinician’s office and just never go back because they’re not good enough. [P3011]* |
|  | *…I was just wondering could you - if you weren’t - if you didn’t agree with something that the doctor said, could you go in and change that and put it in your own words, or not? [P3030]* |
| 1. The IECR brings together an accurate record of relevant information about the service user. | *So they kind of need to actually have a conversation with this new clinician about whether that record’s accurate. How do they feel about the record? Is there anything that they want changed on the record because it’s wrong? [P3011]* |
|  | *Question 4: How might Riley react to her psychiatrist admitting to recording less detailed information in Riley's IECR?*  *Answer:*  *Concerned that the information in the IECR doesn’t truly reflect their sessions. Not a true picture of the situation so unreliable Riley would feel they had not been all together truthful. [P3090]* |
| 3.1: Care professionals document information in the IECR that balances the need for care and the risk to service users’ privacy. | *Yeah, only the relevant info needs to be…info that's connected with the treatment, that's all I wanted to state there. Or info that could incur other understandings for other service providers to treat the client or patient. I wouldn't put records like if a person talked about his dog and his house and that, I wouldn't put information if he’s talked about his birthday, what he did in his birthday…. there has to be a guideline to what information can be loaded on there. You can't sort of put everything that that person said, you know, or you can't put, you know, social things that that person said. [P3010]* |
|  | *Some specific statements were made and the clinician changed it to something diluted. If there’s anything less than that in the medical records, there are some patients that feel they weren’t being heard, and they would want to rely on the records to be able to contest their treatment program. [P3070]* |
| 3.2: The IECR is a reliable and accurate record according to the service user. | *Well, as I spoke earlier, because a lot of service providers put their own records in there, their own perceptions of what the dialogue was, so there would be a diverse way of writing records and in that diversity, there may be a chance that inaccuracy can arise...Also because, yeah, sorry, because it's done quick and rapid, the chances of an inaccuracy occurring can be high...obviously it impacts their health, it could damage – it could do harm to the client. [P3010]* |
|  | *I have had a situation for many years where I was a heavy drinker and alcoholic, and, having gone through counselling and being sober…I still think that’s something in my past that I can be quite judged for now, where my life is completely different. I’m always mindful that when I’m starting a new service…that is in my medical records and, in a lot of ways, it’s okay, because that is relevant…and things like that I went through…alcohol and drug counselling. [P3090]* |
| 1. Service users are empowered to confidently use their IECR | *Finally, he needs to be in receipt of information that indicates how he can check at any time, the effectiveness of his settings, such that he may alter the settings, after accessing information that shows the history of successful and failed access thus far. [P3070]* |
|  | *Well, firstly I would have to say that, for me, it would be a benefit in that with my memory, in relation to my depression, I can go to the doctor and not even remember that afternoon that I’ve been, it’s gone from my brain…which means that then I’ve got holes in explaining what my medications are, or what works for me...[P3060]* |
| 4.1: Service users receive detailed information about their IECR. | *Just the fact that it’s a not opt-in, it’s an opt-out, to me, that says I don’t want a bar of it. I should be allowed to have the choice and say okay. But if I - if I’m not well or something or I forget or I switch and the personality that’s running around doesn’t know, well it’s automatically gone ahead and I haven’t been able to opt out of it. I don’t like that at all. [P3011]* |
|  | *Riley would need an advocate to explain to them, what they needed to know, because I think it needs to be summarised verbally, and it would be overwhelming if it was provided in written form. [P3070]* |
| 4.2: Service users are supported to use their IECR. | *Yeah, when you're psychotic, which occurs occasionally when you're schizophrenic, you don't have a sufficiently coherent view of the world and its operations to be able to consent to really anything very much. Anything as important as deciding the fate of medical records, or any details that might have legal implications, you just don't have a capacity to consent to until you are over your psychotic state of mind. [P3070]* |
|  | *He should be considering that he is not educated or equipped with sufficient knowledge of setting privacy settings, to be able to undertake such a critically important software task. In the absence of such knowledge, he needs to be provided with adequate instructions that indicate the potential liabilities and implications of every possible variation in the settings he is in charge of. He should also be informed about the importance of being provided with the opportunity to set exclusions in order to prohibit specific entities (organisations / organisation types) from accessing it. Finally, he needs to be in receipt of information that indicates how he can check at any time, the effectiveness of his settings, such that he may alter the settings, after accessing information that shows the history of successful and failed access thus far. [P3070]* |
| 1. Care professionals use the IECR to proactively support and coordinate care across all services. | *I think because everyone is so different, it just depends on their current situation. I would probably choose to have the record, but also have the knowledge that I’m still likely going to have to be repeating a lot of things and discussing what’s relevant. What’s the issue at hand, and not too much trust in the system that they are going to consider the record. [P3080]* |
|  | *Question 2: What is your opinion on an integrated electronic care record for Jona? What might be the benefits and risks of such a record?*  *Answer:*  *Benefits could be:*  *Cost Saver for Jona & Service Provider*  *Time Saver for both*  *Avoid repetition of Work*  *Avoid adverse Drug events*  *Enhanced Patient Self Management*  *Improvements in Patient Outcomes*  *Reduce Time in gathering relevant Info*  *Avoid duplication of Services*  *Save on Space, Filling cabinets, Paperwork*  *Convenience & Efficiency*  *Patient Accessibility allows Patient to review entries. [P3010]* |
| 5.1: The IECR supports collaborative and integrated care across all services. | *IECR could well pick up on Jonas new-found isolation so that steps could be taken to help him ease into both new social circumstances and his new team of clinicians and support workers. [P3011]* |
|  | *The benefits include…better quality care, through better informed treatment…an opportunity for professional service providers and their representatives to identify areas of need, inconsistency, inadequacy or overlap, facilitating improvement in the provision and cost effectiveness of health care service provision…[P3070]* |
| 5.2: The IECR supports more proactive care and feedback loops between services. | *it would be useful - if you’re presenting and you’re incapable of communication then at least they can see what medications you’re on…if I’m having an acute episode then I have to go to the…hospital, and one time I went they didn’t know what medication I was on, they didn’t know my diagnosis, so, it was like a stab in the dark…and put me on medication, which wasn’t the medication that I’d been taking. If they’d had a record then they would then at least know, oh, well she’s on this, this, and this, and please do not give her any antidepressants because you’ll make her go manic, you know. [P3060]* |
|  | *I see a good point for having non-clinical on there as well because a lot of people, their recovery is based on mostly non-clinical interventions. The clinical interventions are seen as just sharp, short and simple but the non-clinical are more long term and most of the recovery, I believe anyway to a certain extent, is done through non-clinical. [P3010]* |
| 5.3: The IECR supports service users in retelling their story. | *It is likely that it will not be a smooth transition, even with the treating team having access to the care record. Although the case worker will have access to the record, there is always additional information that is needed. Furthermore, sometimes the information can be misinterpreted and bar the client from accessing NDIS because no further clarification is sought. There is an expectation that there will be some gaps in information. [P3080]* |
|  | *…not having to retell my story, not having to be like, hey, so I've received this list of services, not having to use brain space to remember all of those things when I'm asked for a history. [P2030]* |
